# Integrated analysis of miRNAs and mRNAs in thousands of single cells

**DOI:** 10.1038/s41598-025-85612-z

**Published:** 2025-01-10

**Authors:** Jia Li, Jing Tian, Tao Cai

**Affiliations:** 1 National Institute of Biological Sciences, Beijing, China; 2 Tsinghua Institute of Multidisciplinary Biomedical Research, Tsinghua, China

**Keywords:** Transcriptomics, RNA sequencing

## Abstract

The simultaneous sequencing of multiple types of biomolecules can facilitate understanding various forms of regulation occurring in cells. Cosequencing of miRNA and mRNA at single-cell resolution is challenging, and to date, only a few such studies (examining a quite limited number of cells) have been reported. Here, we developed a parallel single-cell small RNA and mRNA coprofiling method (PSCSR-seq V2) that enables miRNA and mRNA coexpression analysis in many cells. The PSCSR-seq V2 method is highly sensitive for miRNA analysis, and it also provides rich mRNA information about the examined cells at the same time. We employed PSCSR-seq V2 to profile miRNA and mRNA in 2310 cultured cells, and detected an average of 181 miRNA species and 7354 mRNA species per cell. An integrated analysis of miRNA and mRNA profiles linked miRNA functions with the negative regulation of tumor suppressor and reprogramming of cellular metabolism. We coprofiled miRNA and mRNA in 9403 lung cells and generated a coexpression atlas for known cell populations in mouse lungs, and detected conserved expression patterns of miRNAs among lineage-related cells. Based on this information, we identified informative age-associated miRNAs in mouse and human lung cells including *miR-29*, which can be understood as a conserved marker for immunosenescence. PSCSR-seq V2 offers unique functionality to users conducting functional studies of miRNAs in clinical and basic biological research.

## Introduction

miRNAs and other forms of small RNAs are essential regulators of many biological processes and diseases^1^, and miRNAs participate in mRNA posttranscriptional regulation by accelerating target mRNA degradation or repressing target mRNA translation^1–4^. Accordingly, it is expected that miRNA and mRNA cosequencing will facilitate the understanding of miRNA functional mechanisms. Current sequencing technologies can generate multidimensional genomics information from single cells, which has facilitated our understanding of the cellular status and molecular relationships of individual cells. The single-cell miRNA and mRNA cosequencing studies published to date have used “lysis and splitting” methods, in which the lysed cell contents are split into two aliquots for miRNA and mRNA sequencing^5^ or polyadenosine (PolyA) enriched beads are used to extract the mRNA content for mRNA sequencing. Then, the remaining RNA contents are used for small RNA sequencing^6^. However, “lysis and splitting” methods are limited by scale and make it difficult to analyze tissue samples.

The recently reported “Total-RNA-seq” methods^7,8^ can capture coding and noncoding genes (including miRNAs) together in one tube, but the miRNA content analyzed is sparse (generally less than 0.5% of total sequencing reads in cultured cells). Thus there is no practical way of coprofiling miRNA and mRNA expression in tissues at single-cell resolution. To address these limitations, we developed a parallel small RNA and mRNA coprofiling method (called PSCSR-seq V2), which can generate small RNA libraries and mRNA libraries simultaneously in many cells while preserving cell identity using barcoding sequences. We used PSCSR-seq V2 to analyze the regulation between miRNA and mRNAs in cultured cells. We also investigated aged-associated miRNAs in lung tissues at single-cell resolution, and ultimately found that *miR-29* can be understood as a conserved marker for immunosenescence in mice and humans.

## Results

### Development of PSCSR-seq V2

**Fig. 1:**
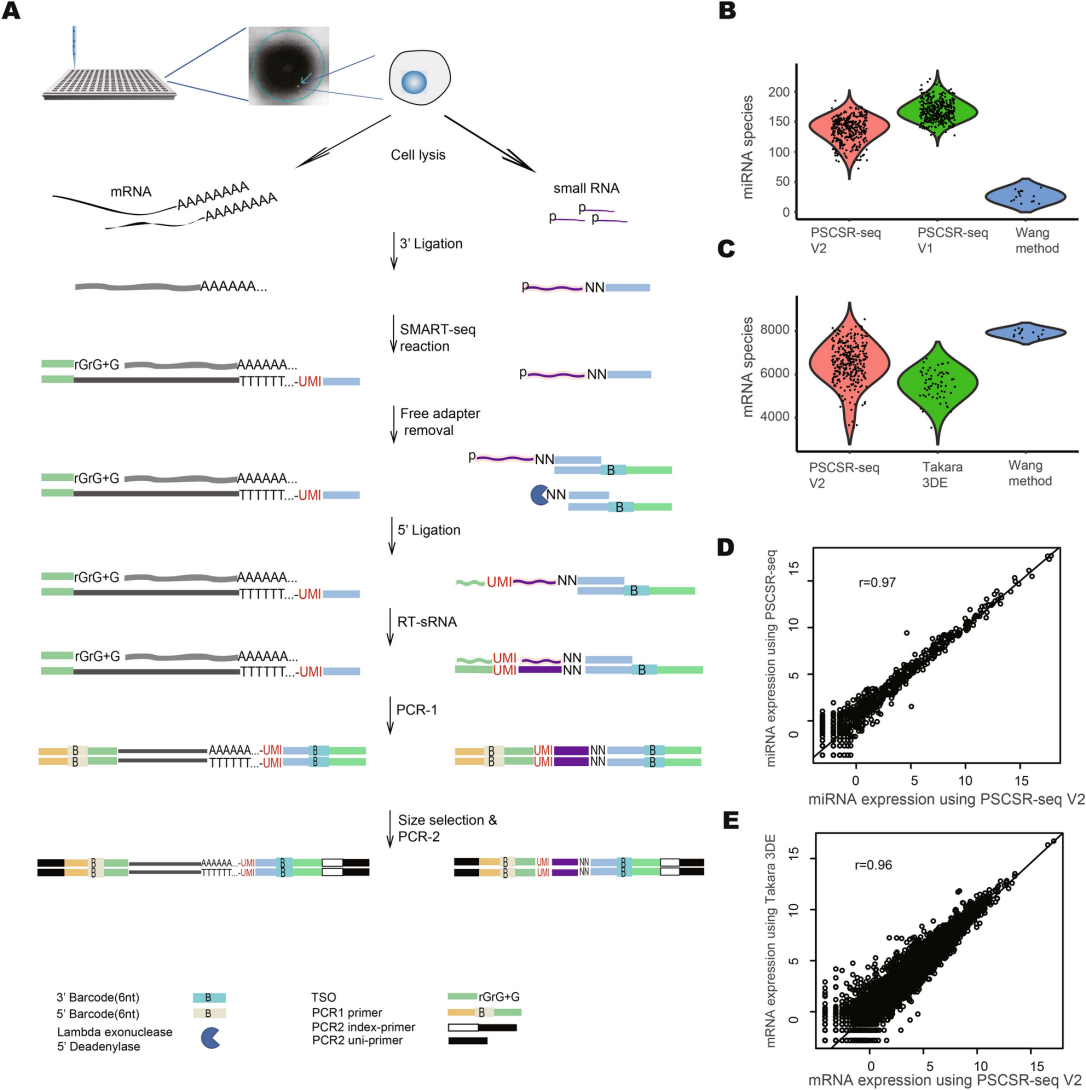
High-quality single-cell small RNA and mRNA cosequencing using PSCSR-seq V2. (**A**) Flowchart of PSCSR-seq V2. Single-cell suspensions were dispensed into a nanowell chip for cell imaging and screening. Selected single live cells were lysed to release total RNA; then, small RNAs were ligated with 3’ adapters, and mRNAs were reverse transcribed and linked with the adapter using a template switching oligo (TSO). Next, the remaining 3’ adapters were removed, and 5’ adapters were ligated to small RNAs. The ligated small RNAs were then reverse-transcribed. Cell barcodes were introduced by PCR to label individual cells (PCR-1). Next, small RNA and mRNA libraries were separately collected. Then, the small RNA and mRNA libraries were amplified for high-throughput sequencing (PCR-2). (**B**, **C**) Comparison of existing methods. Violin plots showing the distribution of miRNA species number per cell (**B**) and the mRNA species number per cell (**C**) detected by the indicated methods (comparing PSCSR-seq V2 and the Wang method; the PSCSR-seq V1 and Takara 3DE sequencing methods were used as references for standalone miRNA and mRNA comparison separately). In the comparison, we sampled 100 K reads for small RNA library analysis and 200K reads for mRNA library analysis. (**D**, **E**) Correlation of miRNA (**D**) and mRNA (**E**) normalized expression (log2_RPM, reads per million library size) in aggregated profiles of K562 cells from PSCSR-seq V2 and reference methods. Pearson correlation coefficients were presented.

The early single-cell small RNA sequencing methods suffer from limitations in efficiency and scale; we previously developed PSCSR-seq^9^, a highly sensitive and efficient method that enables single-cell small RNA profiling for thousands of cells including for clinical biopsies. For the PSCSR-seq V2 method developed in the present study, we retained the same general approach as PSCSR-seq for the small RNA library construction step (see Fig. 1A and Methods), but we added a SMART-seq reaction to enable mRNA library construction (adapted from a commonly used single-cell 3’-mRNA sequencing protocol^10^). In PSCSR-seq V2, adapter ligation for small RNAs and mRNAs are key enabling steps, as ligation efficiency is expected to strongly affect the success of library construction for both small RNA and mRNA. Note that the small RNA 3’ adapter ligation (ligating a DNA adaptor to small RNA) is performed at the very beginning. The mRNA is reverse transcribed and linked with adapters using a SMART-seq reaction, capturing the mRNA information and achieving barcode labeling. The standard PSCSR-seq method is then used for small RNA library construction.

This sequence of steps enables library construction for both small RNA and mRNA in one tube. Because of the different lengths of small RNA and mRNA molecules, a size selection step is used to separate the small RNA library, the mRNA library, and adapter dimers. Then, the libraries are sequenced separately (see Methods). In our design, cells are demultiplexed during data analysis based on their dual-indexed barcode sequences, and the miRNA and mRNA expression levels are estimated using unique molecular identifier (UMI) counts.

### Comparing PSCSR-seq V2’s performance to standalone single-cell small RNA/mRNA methods and “lysis and splitting” methods

We evaluated the performance of PSCSR-seq V2 against low throughput “lysis and splitting” methods and against standalone single-cell small RNA/mRNA methods. Examining K562 cells, we first compared the small RNA profiles from two coprofiling methods (PSCSR-seq V2 and the recent Wang method^5^, with reads at the same depth to enable comparison). In the Wang method, half of the K562 cell sample content is used for small RNA sequencing, and the detected miRNA species number per cell in our hands was 26 ± 9 (miRNA precursor number, mean ± sd). For PSCSR-seq V2, we detected 140 ± 21 miRNA species per cell, a 5.3-fold increase over the Wang method (Fig. 1B). PSCSR-seq V2 detected a lower number of miRNA species than the standalone PSCSR-seq V1 (167 ± 16). To confirm that the additional SMART-seq reaction has a minimal influence on the relative miRNA expression levels, we pooled cells together and compared the correlation of aggregated expression from PSCSR-seq V1 and PSCSR-seq V2, the result shows that the expression is very well correlated (Pearson correlation coefficient = 0.97, Fig. 1D; also see Supplementary Fig. S2).

In the Wang method, the remaining half of the K562 cell sample is used for the mRNA expression analysis, based on a single-cell SMART-seq protocol. In our hands, this method detected 7,927 ± 158 mRNA species per cell (gene number, mean ± sd), which is 18% higher than the value for PSCSR-seq V2 (6,483 ± 864). PSCSR-seq V2 achieves performance at the level of a nanowell-based single-cell 3’-mRNA profiling method; for example, Takara 3DE sequencing^11^ detected 5,556 ± 657 mRNA species per cell (Fig. 1C), and the aggregated mRNA expression level is highly consistent with our result (Pearson correlation coefficient = 0.96, Fig. 1E; see Supplementary Fig. S3 for the comparison between PSCSR-seq V2 and the Wang method). We subsequently assessed PSCSR-seq V2 for applications in cultured cells and tissue samples.

### Integrated analysis of miRNA and mRNA profiles in cultured cells

**Fig. 2:**
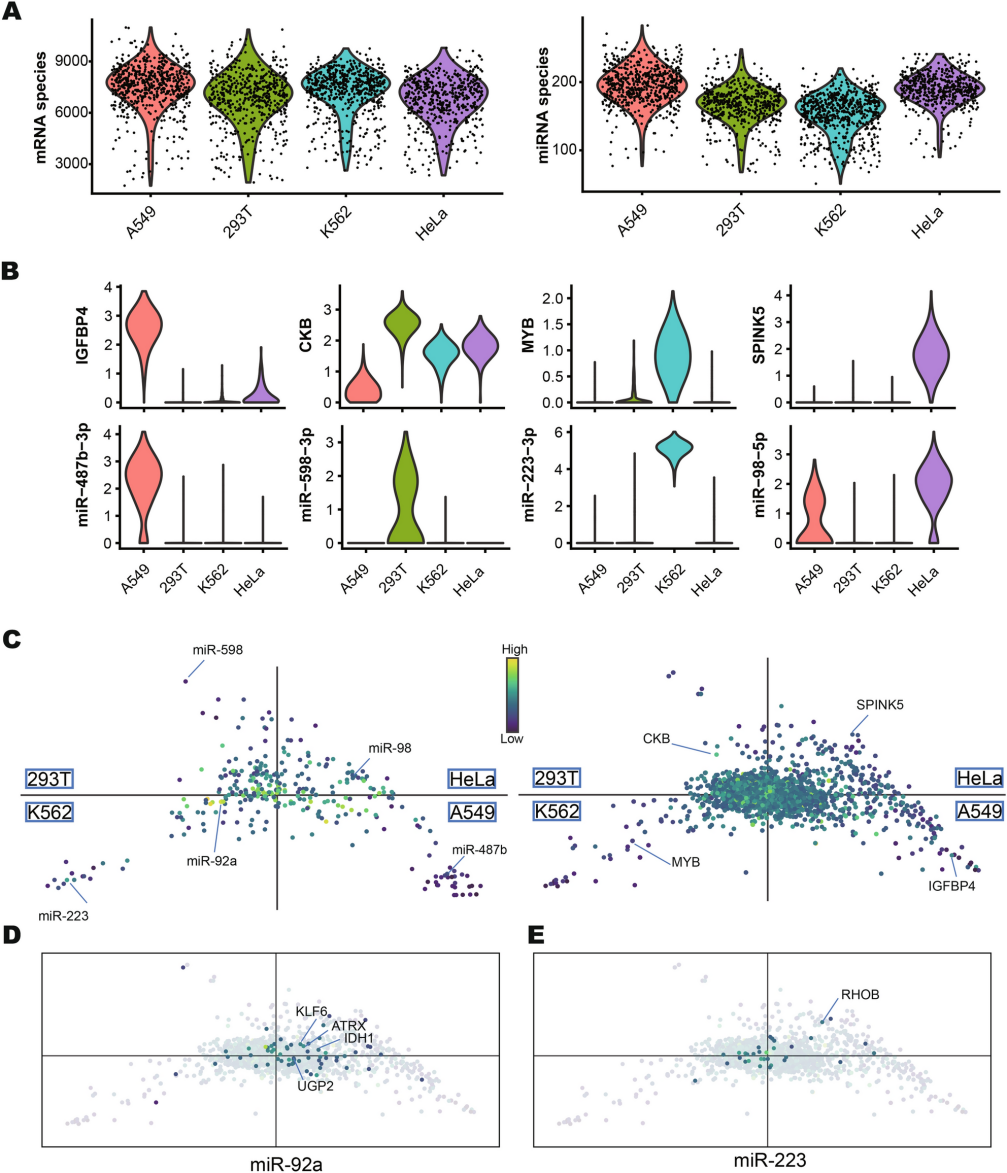
Single-cell analysis of small RNA and mRNA coprofiles in cultured cells. (**A**) Violin plots showing the distribution of miRNA species number and the mRNA species number of individual cells (N = 625 for A549 cells, N = 539 for 293T cells, N = 611 for K562 cells and N = 535 for HeLa cells). (**B**) Violin plots showing the expression of cell specific miRNAs and mRNAs. (**C**) Coinertia analysis of miRNAs and mRNA expression profiles. The miRNA and mRNA expression profiles from different cell types are averaged, then projected into a 2D space (left, miRNA space; right, mRNA space). Coinertia analysis reveals the coordinates that are maximally similar between miRNAs and mRNAs. The cell specific miRNAs/mRNAs are indicated in the plot. (**D**, **E**) The distribution of target mRNAs in mRNA space is highlighted (**D** for *miR-92a* and **E** for *miR-223.* The uncolored points represent the distribution of non-targeted genes). The positions in the 4-quadrant space reflect the relative expression patterns of the mRNAs in 4 cell types, and the colors show the expression levels (dark for low; light for high). The targets with apparent functional relevance are indicated. Please visit the webpage (https://biocaitao.github.io/PSCSRII) for the interactive coinertia analysis.

We first performed coprofiling of small RNA and mRNA in a sample comprising a mixture of cultured cells (A549, K562, HeLa, and 293T). We examined 2,359 individual cells, generating 857M and 1317M raw reads for small RNA and mRNA, respectively. There were 2,310 cells for which both small RNA and mRNA data were commonly captured. For each cell, an average of 134 K small RNA reads and 354 K mRNA reads were mapped to the genome. Similar to the case with PSCSR-seq, miRNAs are the major component of the small RNA library (~ 55% of small RNAs captured are miRNAs, see Supplementary Table S1). An average of 181 miRNA species and 7,354 mRNA species were detected per cell (Fig. 2A). Known cell specifically expressed miRNA and mRNA are presented in Fig. 2B. Unsupervised clustering analysis supported that the four constituent cell types were readily distinguishable based on the miRNA data and based on the mRNA profiles (Supplementary Fig. S1).

miRNAs can downregulate the expression of target mRNAs by promoting the RNA degradation. To visualize the correspondence between miRNAs and their target mRNAs, we applied a multivariate analysis approach known as coinertia analysis^12,13^, which projects distinct datasets (of divergent dimensionality) onto the same coordinates to support the exploration of trends and relationships among different features in those datasets. The miRNA and mRNA profiles were projected to a 2D plane, with each quadrant representing the miRNAs and mRNAs specifically expressed in each of the four cell types (Fig. 2C, the cell specific miRNA and mRNA are highlighted in the plots). For example, *miR-92a* and *miR-223* were highly expressed in K562 cells (located in the K562 quadrant of miRNA space, Fig. 2C), while *miR-92a* mRNA targets were enriched in both the A549 and HeLa quadrants (mRNA space, Fig. 2D), whereas the *miR-223* targets were enriched in both the K562 and 293T quadrants (mRNA space, Fig. 2E). These observations reflected that multi-scale factors (including transcription, processing, and degradation among others) regulate the mRNA expression.

mRNA measurements showed that the *RHOB* gene (a member of the Ras homolog gene family) was downregulated in K562 cells (mRNA space, Fig. 2D). *RHOB* is a validated target of *miR-223* and a known suppressor of tumor transformation, invasion, and metastasis^14^, our result linked *miR-223* expression with the negative regulation of tumor suppressor in K562 cells. Similarly to *miR-223*, *miR-92a* targets genes including *ATRX*^15^ (a chromatin remodeling gene) and *KLF6*^16^ (a zinc finger transcription factor), both of which are candidate tumor suppressors that we found to be downregulated in K562 cells, indicating negative regulation by *miR-92a* in tumor suppression. Further exploring the *miR-92a* targets, *IDH1* (an isocitrate dehydrogenase enzyme in the tricarboxylic acid cycle) and *UGP2* (a UDP-Glucose Pyrophosphorylase) were downregulated in K562 cells, linking *miR-92a* expression with the reprogramming of cellular metabolism^7^. Using cointeria analysis, we can efficiently explore miRNA and mRNA target expression distributions, which should facilitate studies of miRNA functional mechanisms.

### Integrated analysis of miRNA and mRNA profiles in mouse lung tissue

**Fig. 3:**
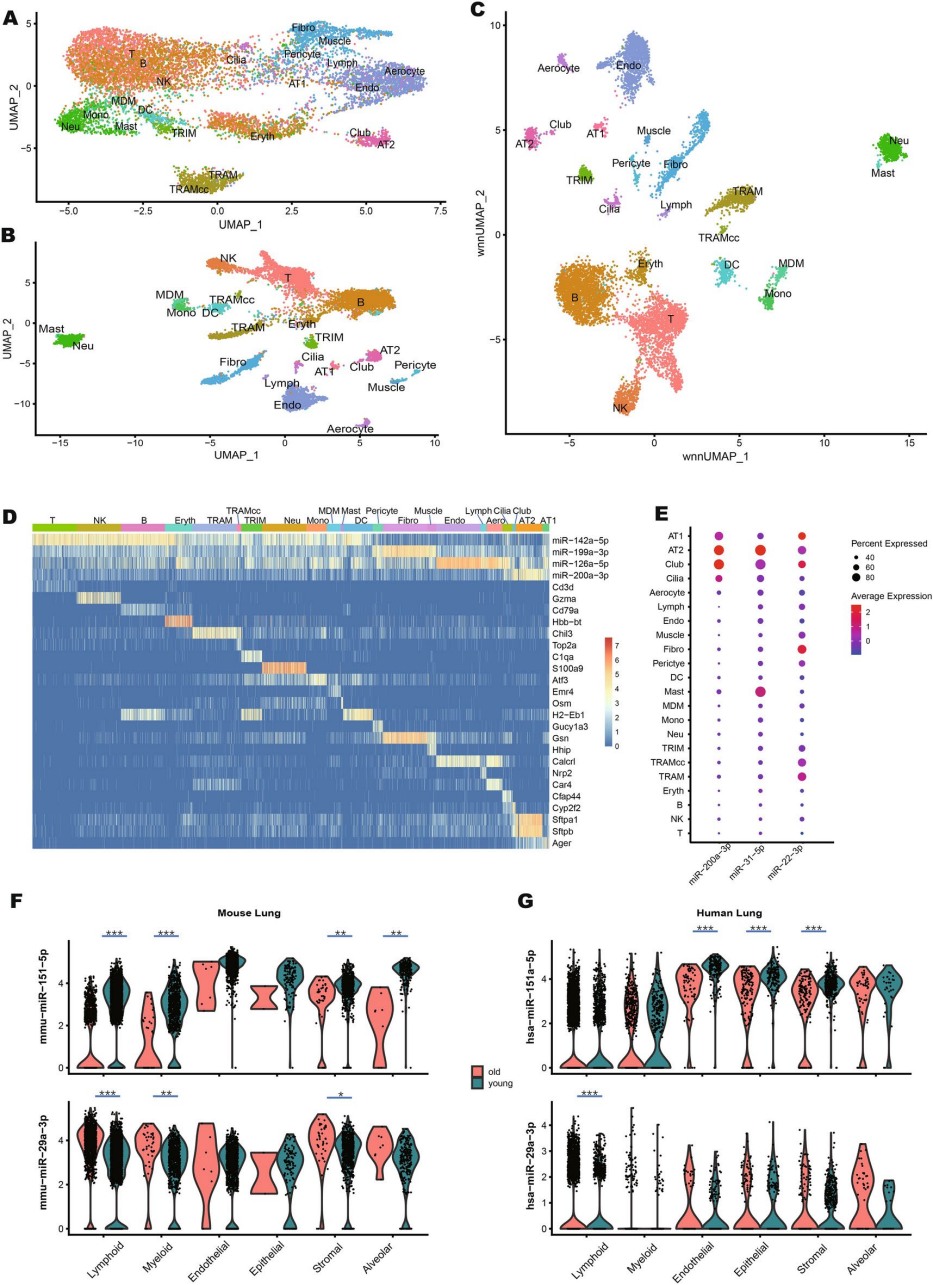
Atlas of miRNA and mRNA coexpression in lung cells. (**A**) UMAP plot of miRNA expression profiles of 9403 cells from young or old mouse lungs. (**B**) UMAP plot of mRNA expression profiles of same lung cells. (**C**) Integrated plot of miRNA and mRNA profiles using the weighted nearest neighbor (WNN) algorithm^18^. (**D**) Heatmap showing the expression of conserved miRNAs among cell types (cell type mRNA markers are presented below). miRNAs are conserved among lineage-related cells, e.g., *miR-142* is expressed in multiple immune cell types; *miR-200* is expressed in epithelial cells; *miR-126* is expressed in endothelial cells; *miR-199* is expressed in stromal cells. (**E**) Dot plot showing combined miRNAs (*miR-200a*, *miR-31*, and *miR-22*) can distinguish alveolar cells. Cell types are listed on the y-axis. miRNAs are listed on the x-axis. Dot colors reflect the average expression level (scaled across cell types), and dot sizes represent the percentage of cells of each cell type that express the indicated miRNAs. (**F**, **G**) Single-cell miRNA expression comparisons between old and young lungs (F, mouse lung and G, human lung; see Methods). The y-axis is for the normalized miRNA expression, and the x-axis is for cell subpopulations. *** for multiple-test adjusted (Bonferroni correction) *p* value < 0.001, ** for adjusted *p* < 0.01, * for adjusted *p* < 0.1. TRAM, tissue-resident alveolar macrophages; TRAMcc, TRAMs in the cell cycle phase “G2M”; TRIM, tissue-resident interstitial macrophages; Neu, neutrophils; Eryth, erythroid cells; Mono, monocytes; MDM, monocyte-derived macrophages; DC, dendritic cells; Endo, general capillary endothelial cells; Fibro, fibroblasts; AT1, alveolar type 1 cells; AT2, alveolar type 2 cells; Lymph, lymphatic endothelial cells; Aero, aerocytes.

Standalone single-cell small RNA sequencing analysis suffers from the lack of high-resolution expression atlases, making it difficult to annotate cells in heterogeneous samples. PSCSR-seq V2 can overcome this limitation by leveraging existing mRNA expression atlas information. To substantiate this, we generated a miRNA/mRNA coexpression atlas database for mouse lung tissues, based on analysis of 7,798 cells from young (2, 3, 5 months) mice and 1605 cells from aged (30 months) mice (the database can be explored at the website https://biocaitao.github.io/PSCSRII). We detected an average of 104 miRNAs and 859 mRNA species per cell. Using the mRNA data and unsupervised clustering method, we annotated 22 cell types, including erythrocytes, alveolar cells, fibroblasts, pericytes, aerocytes, and capillary cells (Fig. 3). This annotation can support various investigations into miRNA functions. For example, previous bulk sequencing showed *miR-200a* expression in mouse lungs^17^, and our PSCSR-seq V2 results indicated that *miR-200a* is expressed most extensively in alveolar cells and ciliated cells (both are epithelial cell types, Fig. 3D). Additionally, we cataloged the miRNAs specifically expressed in the various epithelial cell types (*e.g.*, alveolar type 1 and type 2 cells; see Fig. 3E).

Uniform manifold approximation and projection (UMAP) plots were constructed to visualize the distinct patterns of miRNA and mRNA profiles in mouse lung cells (Fig. 3A,B). In the plots, lineage-related cells contained diverse mRNA profiles but shared a common miRNA expression signature (Fig. 3D). To measure the global similarity of miRNA/mRNA profiles, we used the RV coefficient from coinertia analysis (range 0 to 1, *i.e.*, from no similarity to completely identical). miRNA and RNA profiles showed considerably different structures (RV = 0.4; less than the protein/mRNA profile similarity, generally 0.5–0.9^13^). Globally, miRNA profile patterns correlated more strongly with cell lineage relationships than with mRNA profiles (Supplementary Fig. S4). We integrated the miRNA and mRNA profiles using the weighted nearest neighbor (WNN) algorithm^18^. The integrated plot had a clearer separation of cell subpopulations than individual miRNA and mRNA profiles (Fig. 3A–C). Thus, combining miRNA and mRNA profiles will facilitate fine-grained classification of cells.

### Single-cell analysis of miRNA effects in aged lungs

Based on the established coexpression atlas, we next explored cell-specific miRNAs in aged lungs. The detected mRNA species number per cell was 641 in old vs. 939 in young, and the miRNA species number per cell was 86 in old vs. 107 in young (Supplementary Table S1). There were also apparent changes in cell identities, including depletion of stem cells and enrichment of immune cells: the lung stem cell (alveolar type 2 cell) proportion decreased from 3% in young lungs to 0.5% in old lungs. The T cell proportion increased from 14% in young lungs to 46% in old lungs. The miRNA expression profiles also changed in old lung cells (see Supplementary Table S4). *miR-151* and *miR-29* family miRNAs (majorly *miR-29a,* Supplementary Fig. S5A, B) are the most consistently differentially expressed miRNAs among different cell types (Fig. 3F and Supplementary Fig. S5C, D).

We ask if the miRNAs from our analysis of mouse lungs are conserved in the aged human lung cells. Specifically, we compared miRNA profiles in 9,533 human lung cells (samples from lung cancer patients aged from 42 years old to 76 years old, see Methods); Among the 7,893 non-tumor cells, expression of *miR-151* decreased consistently in older stromal cells, and expression of *miR-29* increased consistently in older lymphoid cells (Fig. 3G). Recently, a broad survey of noncoding RNA expression in mice tissues found that *miR-29* expression increases with age^19^, but this study lacked cell type resolution. Our single-cell data analysis shows that *miR-29* expression increases with age in multiple cell types of mouse lung, supporting that *miR-29* can be understood as a conserved marker for immunosenescence.

A coinertia analysis showed that the aged lungs were separated from young samples (Supplementary Fig. S6). *miR-151* was reported to target *Ctla4*^20^, a gene that contributes to a decreased ability to fight infection and to a diminished response to vaccination in elderly people^21^. We observed reduced expression of *miR-151* and increased expression of *Ctla4* in the aged mouse lung cells, suggesting that loss of miRNA regulation may be involved in the immune hyporesponsiveness known for aged animals^21^. *miR-29* is thought to target more mRNAs than *miR-151* (see Supplementary Fig. S6), and many *miR-29* targets have been implicated as biologically relevant to the aging process. For example, insulin-like growth factor 1 (*Igf1)* is targeted by *miR-29*^22^, and low expression of *Igf1* contributes to the deregulation of nutrient sensing in old animals^23^.

Among *miR-29* target genes, we found that *Tet3* and *Dnmt3a* were expressed at relatively lower levels in aged vs. young lungs. *miR-29* was reported to regulate the non-CG DNA methylation by targeting *Dnmt3a* in mouse brain^24^, so *miR-29* may influence the epigenetic reprogramming process in aged lungs. Extracellular matrix (ECM) components are known to be dysregulated in aged tissues. *miR-29* is predicted to target the vascular endothelial growth factor gene (*Vegfa*) and vascular collagen genes, such as *Col3a1*, *Col4a1*, and *Col6a3*, all of which participate in the ECM organization. These findings collectively support that increased expression of *miR-29* promotes multiple aspects of aging.

## Discussion

In the present study, we developed a parallel small RNA and mRNA coprofiling method and applied it to a variety of cell and tissue samples. Beyond excellent performance in small RNA profiling, PSCSR-seq V2 provides rich mRNA information that supports miRNA regulation analyses. This attribute of the method enables investigations of miRNAs’ role in (for example) aging procession and analysis of interactions occurring between small RNAs and endogenous RNAs. We generated a coexpression atlas for known cell populations in mouse lungs. In our results, miRNA profiles are less powerful for cell type classification and cell cycle prediction. However, miRNA profiles preserve the lineage relationships, the integration of miRNA and mRNA profiles enables finer-resolved classifications of cells.

RNA expression regulation involves multiple layers of factors. We used coinertia analysis to explore the expression distributions among miRNAs and mRNAs. Coinertia analysis does not intend to integrate genomic profiles at the single-cell level, like “WNN” algorithms, but rather provides an interactive plot illustrating the miRNA and target mRNA expression patterns, which can facilitate the selection of suitable miRNAs and their targets for the following functional studies. The coinertia plot requires a knowledge of miRNA/mRNA target relationships. When this information is unavailable (e.g., with some exogenous RNAs), PSCSR-seq V2 allows correlation analysis across many cells to detect potential interactions between miRNAs and mRNAs.

One caution here is that PSCSR-seq V2 focuses on the transcription level, which does not necessarily reflect the translation status or the final product abundance. Accordingly, additional information such as protein abundance would be helpful for guiding functional interpretations. As PSCSR-seq V2 enables single-cell miRNA and mRNA coprofiling for diverse sample types, we anticipate that this method will have broad applications in clinical and biological research by providing rich information for miRNA functional studies.

## Methods

### Oligonucleotides design and synthesis

The 3’ adapter (RA3-A2N) was obtained from Takara Biomedical Technology; The 5′ adapter (SR5T-UUG) was obtained from Sangon Biotech. Other oligonucleotides were obtained from Sangon Biotech. All oligonucleotides used in this study are described in Supplementary Table S2.

### Cell culture

A549 cells were cultured in DMEM/F-12 (11320082, Gibco, Thermo Fisher Scientific). HeLa and HEK293T cells were cultured in basic DMEM (C11965500BT, Gibco). K562 cells were cultured in basic RPMI-1640 media (C22400500BT, Gibco). All the cultured media were supplemented with 10% (v/v) fetal bovine serum (26140079, Gibco) and 1% penicillin–streptomycin (15140122, Gibco). The cells were cultured at 37 °C in a 5% CO2 humidified incubator. Fresh cells were resuspended with 1 × Dulbecco’s phosphate-buffered saline (DPBS, C14190500BT, Thermo Fisher Scientific).

### Mouse lung tissue dissociation

C57BL/6 WT mice were purchased from Beijing Vital River Laboratory Animal Technology Co., Ltd., and maintained under specific pathogen-free (SPF) conditions with free access to food and water under a 12-h light: dark cycle in IVC cages. Mice were euthanized using carbon dioxide and lung tissues were collected and cut into small pieces (2–4 mm). These pieces were dissociated into single-cell suspensions with PythoN® Tissue Dissociation System following the manufacturer’s instructions (MD1101001, Singleron Biotechnologies). Cell suspensions were added with ACK lysis buffer (A10492-01, Gibco) to lyse the remnant red blood cells, and treated with a Dead Cell Removal Kit (130-090-101, Miltenyi), then filtered through 40-μm plastic mesh (Falcon).

### PSCSR-seq V2 library preparation and sequencing

#### Cell staining and selection

Cell suspensions were stained with a ReadyProbes® Cell Viability Imaging Kit (R37610, Thermo Fisher Scientific) for 20 min, then centrifuged (300 × g, 5 min) and resuspended in 1 × DPBS. After cell counting, the stained cell suspensions were diluted in a mix of 1 × Second Diluent (640196, Takara) and 0.4 U Ribonuclease Inhibitor (N2515, Promega) to 1 cell/35 nl. The cell suspensions were dispensed into a SMARTer ICELL8 350v Chip (640019, Takara, containing 5184 [72 × 72] nanowells) on a MultiSample NanoDispenser (MSND, Takara). All nanowells of the ICELL8 chip were imaged with a fluorescence microscope (Olympus BX43), and the images were analyzed using CellSelect software (Takara) to determine the viability and number of cells present. Alive single cells were automatically selected with manual checks for the subsequent experiments.

#### Cell lysis and small RNA 3’ ligation

The microchip was frozen in a − 80 °C fridge for at least one hour and transferred to a modified SmartChip thermocycler (Bio-Rad) at 75 °C for 5 min and chilled on ice immediately. 35 nl of 3’-ligation mix containing 0.2% Triton™ X-100 (T9284, Sigma-Aldrich), 0.04 U Ribonuclease Inhibitor, 0.07 pmol 3’ adapter (RA3-A2N, Supplementary Table S2), 0.56 U T4 RNA Ligase 2, truncated KQ (M0373S, NEB) and 2 X T4 RNA ligase buffer was prepared and dispensed into the selected nanowells. The microchip was incubated with a program of 25 °C for 6 h and 4 °C for 8–10 h, followed by 65 °C for 20 min.

#### mRNA reverse transcription

35 nl of fragment buffer (0.07 pmol of mRNA RT primer [MM-T50T, Supplementary Table S2], and 2X RT buffer) was dispensed into the selected nanowells, and heated at 94 °C for 3 min. Next, 35 nl of mRNA RT mix (2 mM dNTP, 0.42 U Maxima H Minus Reverse Transcriptase [EP0753, Thermo Fisher Scientific], 0.07 U Ribonuclease Inhibitor, and 0.14 pmol template switching oligo [TSO-5TL, Supplementary Table S2]) was dispensed and incubated at 50 °C for 90 min followed by 85 °C for 5 min.

#### Small RNA 3’ adapter removal and 5’ adapter ligation

35 nl of miRNA RT primer mix (0.53 pmol barcoded RT primer [SCSR-RTP, Supplementary Table S2], 2.5X lambda exonuclease buffer) was added into selected nanowells, with the program “Index 1” on the MSND. The chip was placed inside a thermocycler at 70 °C for 2 min and then chilled on ice. 35 nl of adapter-removed solution (0.08 U Lambda exonuclease [EN0562, Thermo Fisher Scientific], 0.19 U 5’ Deadenylase [M0331, NEB], 0.04 U Ribonuclease Inhibitor) was dispensed into the microchip, and incubated at 30 °C for 30 min followed by 37 °C for 1 h and then 75 °C for 10 min. Next, 35 nl of 5’-ligation mix (0.07 pmol of the 5’ adapter [SR5T-UUG, Supplementary Table S2], 7 mM ATP, 0.2 U T4 RNA Ligase 1 [M0437M, NEB], 3 X T4 RNA ligase buffer, 0.05 U Ribonuclease Inhibitor) was added into the microchip and transferred to the thermocycler with a program of 25 °C for 2 h and 65 °C for 20 min.

#### Small RNA reverse transcription

The small RNA RT reaction mix (1X First-strand buffer, 48 mM DTT, 1.6 mM dNTP, 0.78 U Superscript III reverse transcriptase [18080-085, Thermo Fisher Scientific], 0.06 U ribonuclease inhibitor) was dispensed into selected nanowells (35 nl of each) and incubated at 52 °C for 60 min and 70 °C for 15 min.

#### PCR barcoding and sample collection

35 nl of PCR-1 mix containing 0.22 pmol barcoded PCR-1 primers (SR5T-P1, Supplementary Table S2), 3.3 X PCR buffer, and 0.01 U Phanta® HS Super-Fidelity DNA Polymerase (P502-d1, Vazyme) was dispensed into the microchip with the program “Index 2”. After dispensation, the microchip was placed in the thermocycler with a program of 95 °C for 3 min, followed by 14 cycles (95 °C for 30 s, 65 °C for 30 s, and 72 °C for 1 min) and a final incubation at 72 °C for 5 min. Finally, the microchip was inverted and centrifuged at 3000 × g for 10 min to collect and pool all contents into a single collection tube using the supplied SMARTer™ ICELL8® Collection Kit (640048, Takara).

#### Purification and library size selection

The collected PCR-1 product was purified twice using 1.8 × SPRIselect Beads (B23319, Beckman Coulter). The size distribution was obtained with an Agilent High Sensitivity DNA Kit (5067–4626, Agilent Technologies) on an Agilent Bioanalyzer 2100 instrument. The quantification was performed using a Qubit™ dsDNA HS Assay Kit (Q32854, Thermo Fisher Scientific). Half of the PCR product was size selected for small RNA library collection using 3% agarose, dye-free, Pippin Prep (CDP3010, Sage Science) at 134–162 bp. The other half was purified with 2% Agarose, dye-free, Pippin Prep (CDF2010, Sage Science) at 270–650 bp to collect mRNA libraries.

#### Library amplification and sequencing

The 50 µl PCR-2 reaction mix was prepared with purified PCR-1 product, 0.1 mM dNTP, 1 X PCR buffer, 1 U Phanta® Max Super-Fidelity DNA Polymerase (P505-d1, Vazyme), 10 pmol SCSR-PCR1 primer (Supplementary Table S2), and 10 pmol SCSR-PCR2 index primer (Supplementary Table S2). The reaction was performed with a program of 95 °C for 3 min, 6–12 cycles of 95 °C for 30 s, 65 °C for 30 s, and 72 °C for 1 min, and 1 cycle of 72 °C for 5 min. The PCR-2 product was purified with 1.6 × SPRIselect beads. The PSCSR-seq V2 library was quantified with a qPCR-based KAPA Library Quantification Kit for Illumina platforms (KK4824, Kapa Biosystems). The PSCSR-seq V2 library was sequenced using an Illumina NovaSeq 6000 System or NextSeq2000 instrument.

Supplementary Table S3 summarizes the experimental steps, as well as their time and reagent costs.

### Bioinformatics analysis

PSCSR-seq V2 generated small RNA libraries and mRNA libraries separately. The small RNA sequences were analyzed using the PSCSR-seq method, which is documented in the literature^9^.

#### mRNA library sequences analysis

The reads index tags, cell barcodes, and inserted sequences were extracted from raw reads using customized scripts (https://github.com/biocaitao/PSCSRII). Then, the inserted sequences were mapped to the genome using the STAR^25^ program with default parameters. The mapped sequences were annotated according to gene features in the GENCODE^26^ database.

To measure the expression of mRNAs, mapped reads with the same index tag or adjacent index tag (1 mismatch) were collapsed into UMI. The UMI counts were weighted by the number of mapped locations, and the UMIs were summed as the measurement of mRNA expression. Then, mRNA expression values for each cell were normalized and log-transformed, implemented using the R “Seurat” package (v4.0.0).

#### Cell clustering analysis

Unsupervised cluster analysis was performed as described in a previous study^27^, and the cluster analysis was implemented in “Seurat”. First, 1000 informative mRNAs were selected based on the “variable stable transform” algorithm. The gene expression profiles were then scaled and projected to the PCA space. The first 15 PCs were selected. The scaled expression profiles were clustered using a graph-based clustering algorithm (the “Louvain” algorithm in Seurat). The resolution parameters were checked from 0.1 to 1 (in 0.1 steps), and we chose a resolution of 0.5 for the data presentation and comparison. The highly expressed mRNAs in each cluster were identified using the Seurat “FindAllMarkers” function with the default settings. The batch effects of different samples were adjusted using the “canonical correlation analysis” method^28^ in Seurat, and The ‘weighted-nearest neighbor’ (WNN) analysis^18^ in Seurat was used to integrate miRNA profiles and mRNA profiles.

#### miRNA-mRNA coinertia analysis

miRNA target mRNAs were predicted using the R “miRNAtap” package^29^; we chose the common prediction in multiple prediction algorithms (N = 3) in “miRNAtap”.

The coinertia analysis between miRNA and mRNA profiles was carried out using the R “MADE4” package^30^. To simplify the presentation, the miRNA/mRNA profiles were averaged across cell types/subpopulations, and the highest expressed miRNAs (n = 200) and mRNAs (n = 2500) were used for the analysis. The global correlation between miRNA and mRNA profiles was measured by the RV coefficient, calculated as the total inertia (sum of eigenvalues of a coinertia analysis) divided by the square root of the product of the squared total inertias (sum of the eigenvalues) from the individual analysis.

#### miRNAs comparison among aged lungs

We compared a 30-month-old mouse lung with three young samples (2, 3, and 5 months old, separately) using PSCSR-seq V2. Wilcoxon rank sum test (implemented in the Seurat package) was used for statistical comparisons. We compared the miRNA profiles from lung adenocarcinoma patients using the PSCSR-seq method^9^. The cells from five patients (ages 42, 57, 63, 70, and 76) were split into two subgroups based on the median age. The human and mouse cells were grouped into six subpopulations according to conserved miRNA markers commonly expressed in human and mouse cells:

*miR-142* and *miR-150* for lymphoid cells including T, B, NK cells; *miR-142* and *miR-223* for myeloid cells including macrophages, monocytes; *miR-126* for endothelial cells including capillary cells, capillary aerocytes; *miR-199* for stromal cells including fibroblast, pericytes or muscle cells; *miR-200* for general epithelial cells such as cilia, and *miR-375* for alveolar cells including AT1, AT2. Tumor cells (with *miR-135b* expression) were removed.

#### Other analysis

The cell cycle phase was predicted using the “CellCycleScoring” function in Seurat.

## Supplementary Information

Supplementary Information 1.

Supplementary Information 2.

Supplementary Information 3.

Supplementary Information 4.

Supplementary Information 5.

Supplementary Information 6.

## Data Availability

The datasets supporting the conclusions of this article are available in the NCBI Gene Expression Omnibus (GEO; https://www.ncbi.nlm.nih.gov/geo/) under accession number GSE226714. Code availability: All source codes are available within the GitHub repository (https://github.com/biocaitao/PSCSRII). Also, the website (https://biocaitao.github.io/PSCSRII) includes related datasets in the paper.

## Code availability

All source codes are available within the GitHub repository (https://github.com/biocaitao/PSCSRII). Also, the website (https://biocaitao.github.io/PSCSRII) includes related datasets in the paper.
