# Supplementary figures and images for: Integrated analysis of miRNAs and mRNAs in thousands of single cells
Source: Sci Rep. 2025 Jan 10;15:1636. doi: 10.1038/s41598-025-85612-z (PMC11724058; doi:10.1038/s41598-025-85612-z)

Figure S1

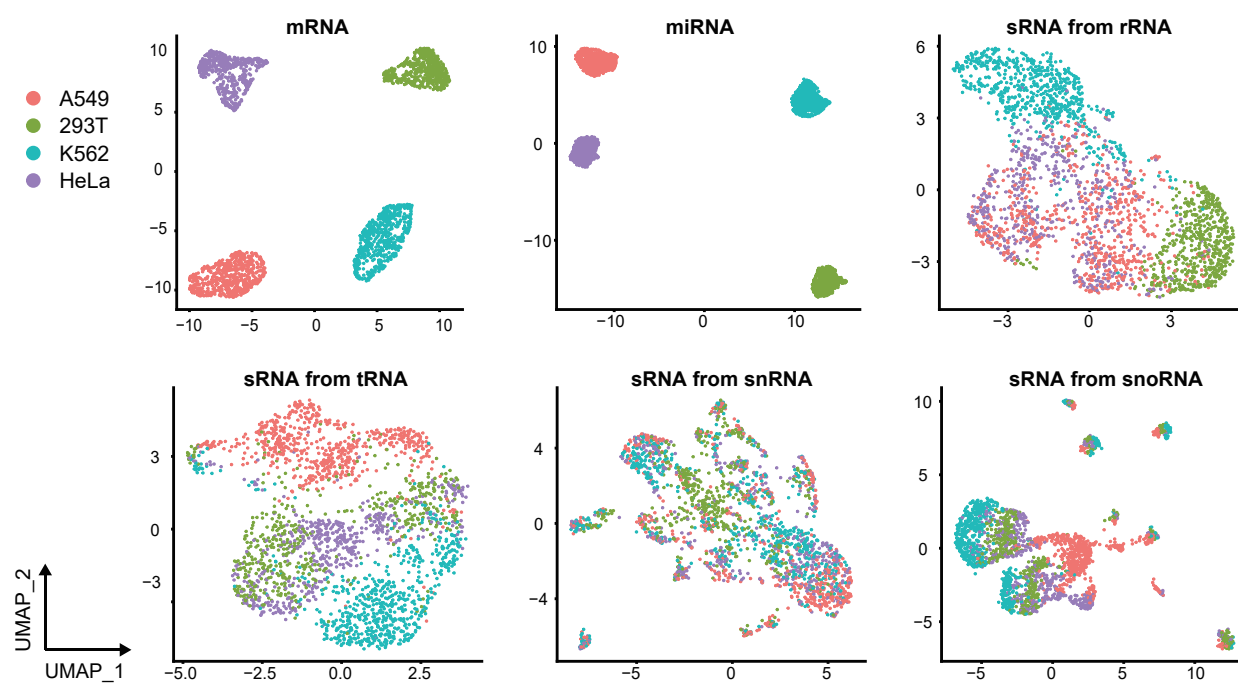

Figure S2

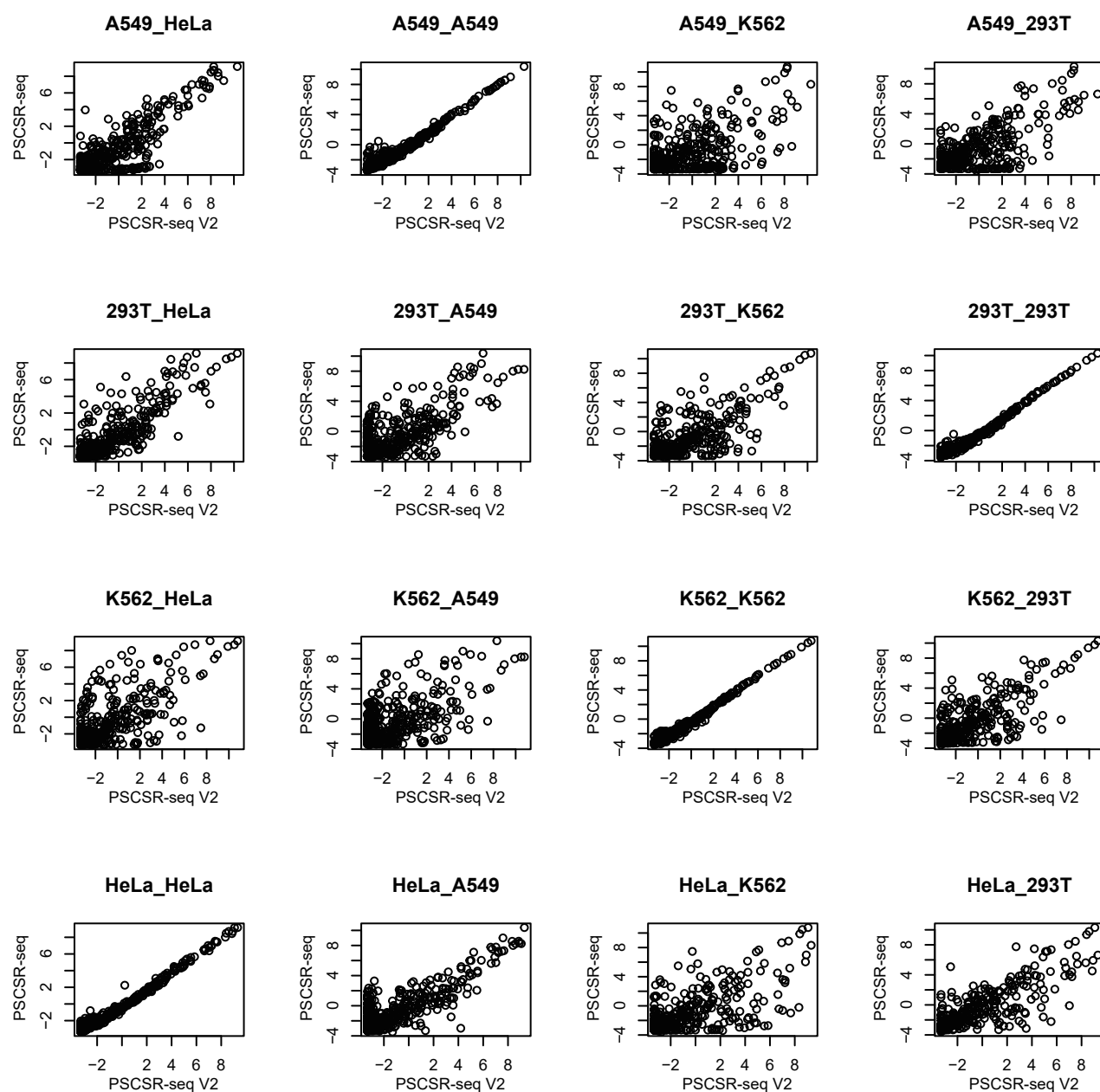

Figure S3

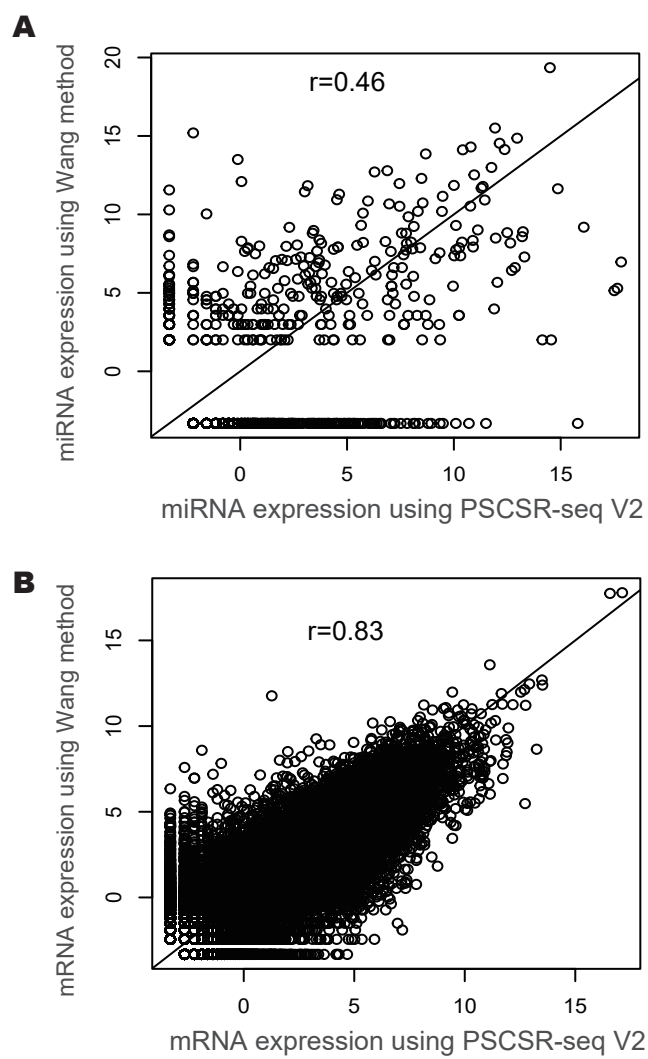

**A**

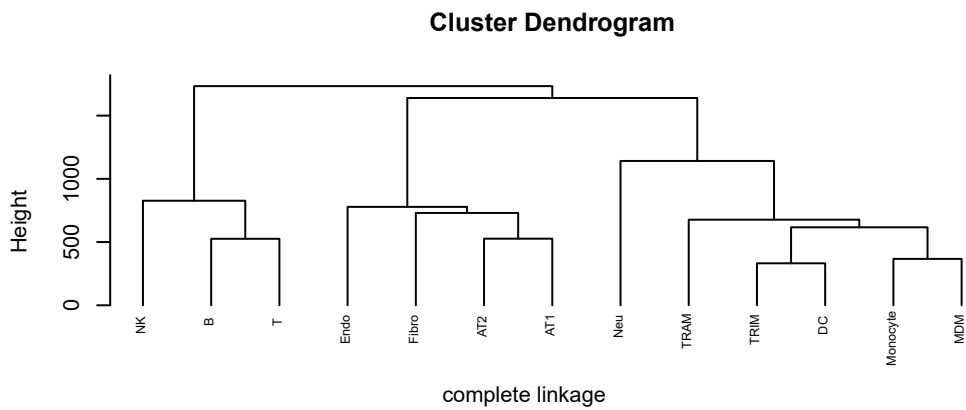

**B**

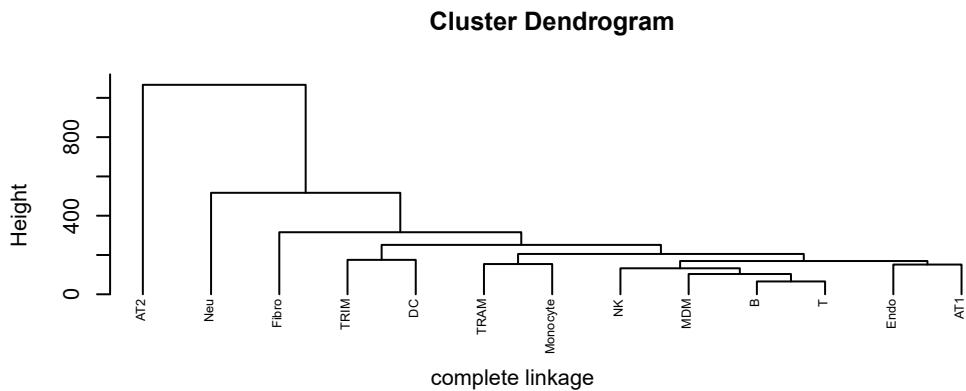

Figure S5

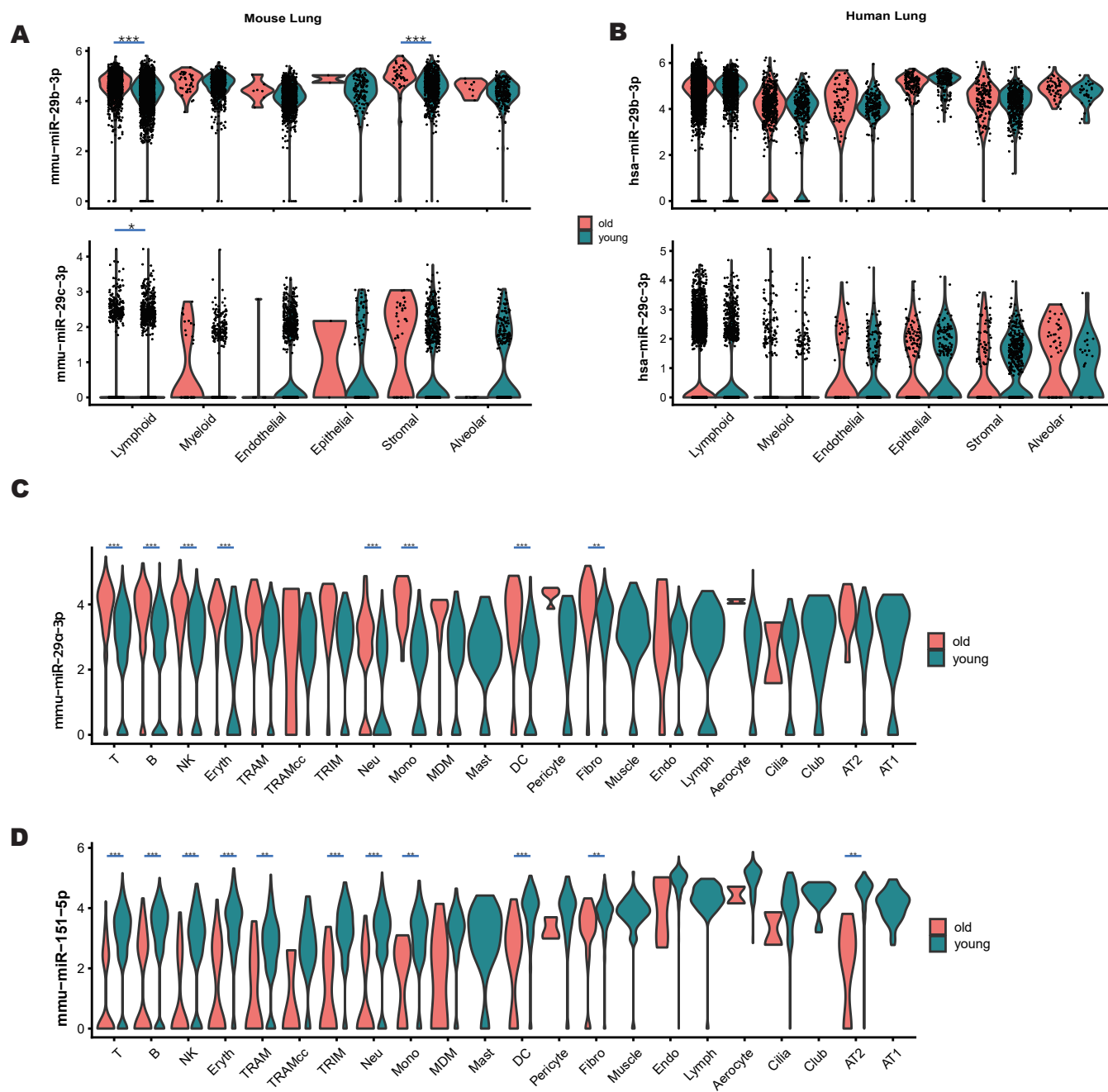

Figure S6

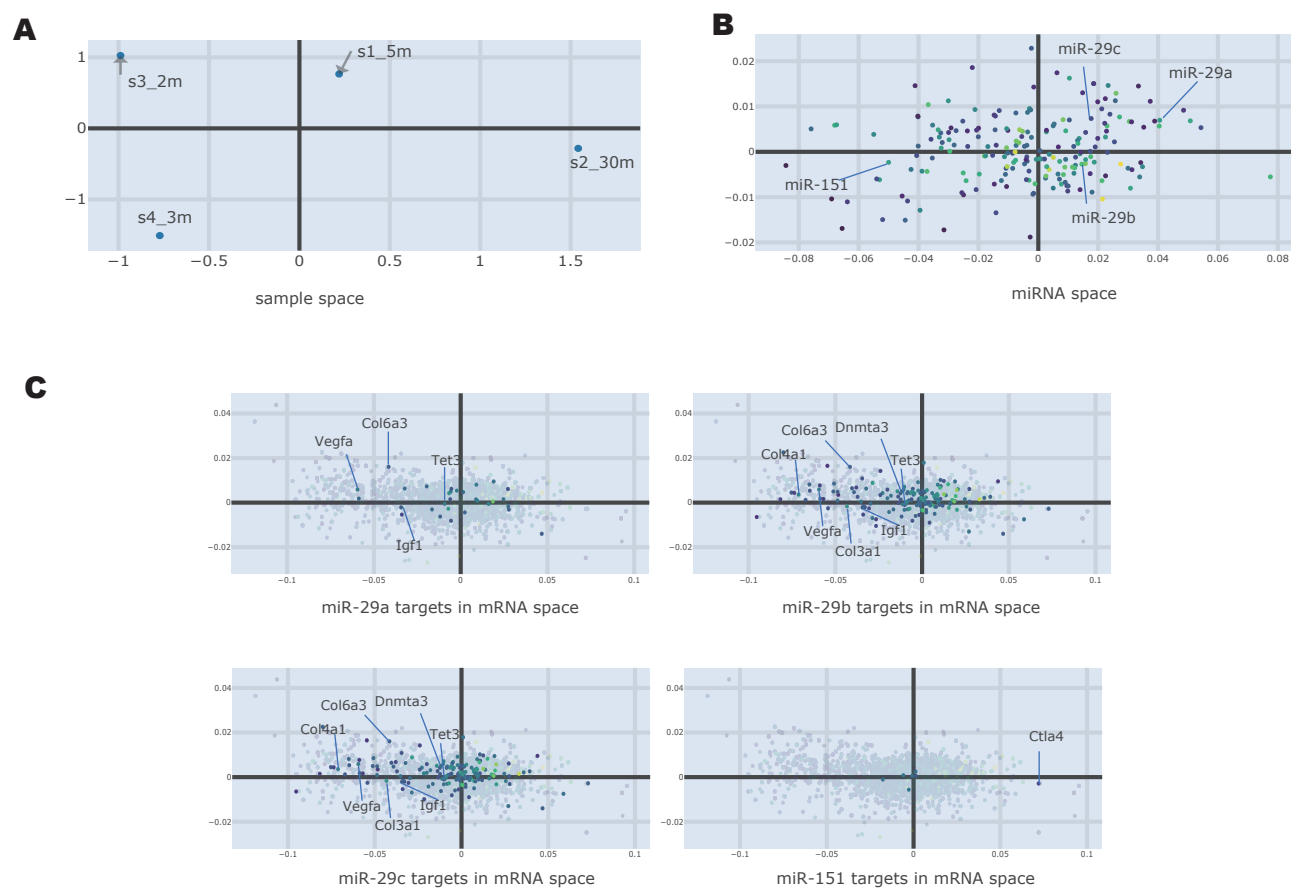

Supplement: Supplementary file 1 — Supplementary Information 1. [file 41598_2025_85612_MOESM1_ESM.pdf]
