# Supplementary material for: Integrated analysis of miRNAs and mRNAs in thousands of single cells
Source: Sci Rep. 2025 Jan 10;15:1636. doi: 10.1038/s41598-025-85612-z (PMC11724058; doi:10.1038/s41598-025-85612-z)
Supplement: Supplementary file 6 — Supplementary Information 6. [file 41598_2025_85612_MOESM6_ESM.docx]

**Supplementary Information (includes Figures S1 to S6, Tables S1 to S4):**

**Fig. S1. Single-cell small RNA and mRNA coprofiling using cultured cells.**

Uniform manifold approximation and projection (UMAP) plots of mixed cells of the indicated lines were calculated from expression profiles of mRNAs, miRNAs, or small RNAs from rRNA, tRNA, snRNA and snoRNA.

**Fig. S2. Comparison of miRNA expression profiles using PSCSR-seq V1 and V2.**

The published datasets^9^ from PSCSR-seq V1 were used for the comparisons. The miRNA expression profiles in each cell type were pooled and averaged. The expression values of miRNAs were adjusted using the canonical correlation analysis (CCA) algorithm in the “Seurat” package. Pairwise comparisons between PSCSR-seq V1 and V2 among cell types were plotted.

**Fig. S3. Comparison between PSCSR-seq V2 and the Wang method**

The reads files from the Wang method^5^ were merged and 2x10^6^ small RNA/mRNA reads were randomly sampled from the Wang method or PSCSR-seq V2. The small RNA/mRNA reads were annotated and counted. Then, the counts were normalized (divided by the sum of all counts and multiplied by 10^6^) and log2-transformed (a constant of 0.1 added before the transformation). The correlations of miRNA (A) and mRNA (B) normalized expression were plotted. The Pearson correlation coefficients were presented.

**Fig. S4. Hierarchical cluster analysis of miRNA and mRNA profiles.**

miRNA (A) or mRNA (B) profiles of major cell populations (epithelial, endothelial, stromal, and immune cells) were pooled and averaged. Then, the Euclidean distances of cell populations were calculated for the hierarchical cluster analysis.

**Fig. S5. miRNA expression in aged lungs.**

(A, B). Violin plots showing the *miR-29b/c* expression difference between old and young lungs (A for mouse lung, B for human lung).

(C, D). Violin plots show the *miR-29a* (C) and *miR-151* (D) expression difference between old and young across all cell types (cell catalogs containing less than 2 cells were dropped).

*** for multiple-test adjusted p value <0.001, ** for adjusted p<0.01, * for adjusted p<0.1.

**Fig. S6. Correspondence between miRNAs and target mRNAs in lung samples**

The miRNA and mRNA expression profiles from four lung samples (ages 2, 3, 5, and 30 months) are averaged and projected into a 2D space using coinertia analysis. Coinertia analysis reveals the coordinates that are maximally similar between miRNAs and mRNAs.

(A) The sample space, arrows indicate the divergence between miRNA profiles and mRNA profiles. (B). The expression distributions of age-associated miRNAs in miRNA space. (C). The expression distributions of predicted targets in mRNA space. Please visit the webpage (https://biocaitao.github.io/PSCSRII) for the interactive coinertia analysis.

Table S1.

Quality control information of cultured cells and mouse tissues

Table S2.

Adapter and primer sequences for PSCSR-seq V2.

Table S3.

Time and reagent cost for PSCSR-seq V2.

Table S4.

The miRNA comparisons between old and young lung cells.
